# In Vitro Antiproliferative and Antioxidant Effects of Extracts from* Rubus caesius* Leaves and Their Quality Evaluation

**DOI:** 10.1155/2016/5698685

**Published:** 2016-12-22

**Authors:** Daniel Mirosław Grochowski, Roman Paduch, Adrian Wiater, Adrianna Dudek, Małgorzata Pleszczyńska, Monika Tomczykowa, Sebastian Granica, Paulina Polak, Michał Tomczyk

**Affiliations:** ^1^Department of Pharmacognosy, Medical University of Białystok, ul. Mickiewicza 2a, 15-230 Białystok, Poland; ^2^Department of Virology and Immunology, Maria Curie-Skłodowska University, ul. Akademicka 19, 20-033 Lublin, Poland; ^3^Department of General Ophthalmology, Medical University of Lublin, ul. Chmielna 1, 20-079 Lublin, Poland; ^4^Department of Industrial Microbiology, Maria Curie-Skłodowska University, ul. Akademicka 19, 20-033 Lublin, Poland; ^5^Department of Organic Chemistry, Medical University of Białystok, ul. Mickiewicza 2a, 15-222 Białystok, Poland; ^6^Department of Pharmacognosy and Molecular Basis of Phytotherapy, Medical University of Warsaw, ul. Banacha 1, 02-097 Warsaw, Poland

## Abstract

The present study was performed to evaluate the effect of different extracts and subfractions from* Rubus caesius* leaves on two human colon cancer cell lines obtained from two stages of the disease progression lines HT29 and SW948. Tested samples inhibited the viability of cells, both HT29 and SW948 lines, in a concentration-dependent manner. The most active was the ethyl acetate fraction which, applied at the highest concentration (250 *μ*g/mL), decreased the viability of cells (HT29 and SW948) below 66%. The extracts and subfractions were also investigated for antioxidant activities on DPPH and FRAP assays. All extracts, with the exception of water extract at a dose of 250 *μ*g/mL, almost totally reduced DPPH. The highest Fe^3+^ ion reduction was shown for the diethyl and ethyl acetate fractions. It was more than 6.5 times higher (at a dose 250 *μ*g/mL) as compared to the control. The LC-MS studies of the analysed preparations showed that all samples contain a wide variety of polyphenolics, among which ellagitannins turned out to be the main constituents with dominant ellagic acid, sanguiin H-6, and flavonol derivatives.

## 1. Introduction 

Drugs of natural origin have been used throughout history to cure or prevent diseases. Modern phytotherapy is engaged in the production of remedies from materials derived from plants and their use in effective and safe therapy. Their main action could be aimed at three aspects: cytostatic activity, especially when therapy concerns tumour tissue, and anti-inflammatory and antioxidative or free radical reduction actions. With all this in mind, we have tried to evaluate the cytotoxic and antioxidant activities of* Rubus caesius* extracts on two human colon cancer cell lines obtained from two stages of disease progression. Additionally, the full phytochemical profile of all the investigated extracts obtained from* R. caesius* leaves based on the HPLC-DAD-MS^*n*^ method has been characterized for the first time.* R caesius* is a well-known shrub (dewberry) extending from Europe to Siberia, but it can also be found in the United States. Folk medicine attributes many virtues to* R. caesius*. Further studies are required to confirm the pharmacological relevance of the findings, but now there are great expectations for its wide therapeutic application [1].

## 2. Materials and Methods

### 2.1. Plant Material and Preparation of Extracts and Their Fractions

The leaves from wild species of* R. caesius* were collected during June-July 2012–2014 from Puszcza Knyszyńska, near Bialystok, Poland. A voucher specimen of plant RC-11027 has been deposited in the Herbarium of the Department of Pharmacognosy, Medical University of Białystok, Poland. All plant samples, extracts, and fractions were prepared according to previously described methods [2]. Yields are as follows: RC1, 83 mg; RC2, 79 mg; RC3, 101 mg; RC4, 9 mg; RC5, 28 mg; RC6, 96 mg.

### 2.2. HPLC-DAD-MS^3^ Analysis

The HPLC-DAD-MS^3^ analysis was performed using similar conditions described previously [2]. HPLC analyses of samples were carried out on a reversed-phase Kinetex XB-C18, 100 mm × 2.1 mm × 1.7 *μ*m column (PHENOMENEX, USA). Compounds were analysed in negative and positive ion modes (the MS^2^ 152, −162, and −176 amu). In the case of the detection of one of the neutral loss masses MS^3^ fragmentation was performed. Analysis was carried out using scan from *m*/*z* 70 to 2.200.

### 2.3. Cell Cultures

Two human colon tumour cell lines were used. HT29 (ATCC® HTB-38™) and SW948 (ATCC CCL-237™) cell lines representing early and late stages of tumour development were cultured as monolayers in 25 mL culture flasks (NUNC, Rochester, USA). All cell lines were maintained in RPMI 1640 medium supplemented with 10% FBS (foetal bovine serum) (v/v) and antibiotics (100 U/mL penicillin, 100 *μ*g/mL streptomycin) (SIGMA, St. Louis, MO, USA) at 37°C in a humidified atmosphere with 5% CO_2_.

### 2.4. MTT Assay

The MTT assay is based on the conversion of a yellow tetrazolium salt by viable cells to purple crystals of formazan. The reaction is catalysed by mitochondrial succinyl dehydrogenase. Cell sensitivity to* R. caesius* extracts was analysed in a spectrophotometric 3-(4,5-dimethylthiazol-2-yl)-2,5-diphenyltetrazolium bromide (MTT) test according to Mosmann [3].

### 2.5. Neutral Red (NR) Uptake Assay

The NR cytotoxicity assay is based on the uptake and lysosomal accumulation of the supravital dye, Neutral Red. Dead or damaged cells do not take up the dye. The method was used as described earlier [4].

### 2.6. Nitric Oxide (NO) Measurement

Nitrate, a stable end product of NO, was determined in culture supernatants by a spectrophotometric method based on the Griess reaction. The course of the procedure has been described previously [5].

### 2.7. DPPH^•^ Free Radical Scavenging Test

The free radical scavenging activity of extracts was analysed by the 1,1-diphenyl-2-picrylhydrazyl (DPPH) assay. The test is based on the ability of antioxidants to reduce the stable dark violet radical DPPH^•^ (SIGMA, USA) to the yellow diphenyl-picrylhydrazine. The methodology has been described in our previous study [5].

### 2.8. Ferric-Reducing Antioxidant Power (FRAP) Assay

The FRAP method was used to determine the antioxidative capacity of the tested extracts. The procedure has been described earlier [4].

### 2.9. Statistical Analysis

The biological experiments were repeated three times. The data were analysed using one-way ANOVA followed by Dunnett's multiple comparison post hoc test. Only results with significance of *p* ≤ 0.05 were considered significant.

## 3. Results and Discussion 

Many species classified to the genus* Rubus* have been recognized as potential agents with significant effects on human health [6–10]. In the present work we selected leaves of blackberry* R. caesius* (dewberry) species traditionally used as a remedy to treat many diseases, among them gastrointestinal bleeding and diarrhoea [1, 11]. More recently, Dudzińska and coauthors indicated that the extracts obtained from dewberry leaves demonstrate antiplatelet activities in whole blood, where neutrophils play a pivotal role in mediating their effects on platelets. Although these extracts do not hamper the neutrophil oxidative metabolism and do not influence the expression of neutrophil adhesive receptors, they demonstrate an ability to lower the reactive oxygen level produced by neutrophils [12]. According to the reviewed literature, little is known about the potential antiproliferative and antioxidant activity of dewberry's leaves which encouraged us to investigate this plant growing in Poland. In addition, there is no solid evidence describing the chemical composition of the species.

**Figure 1:**
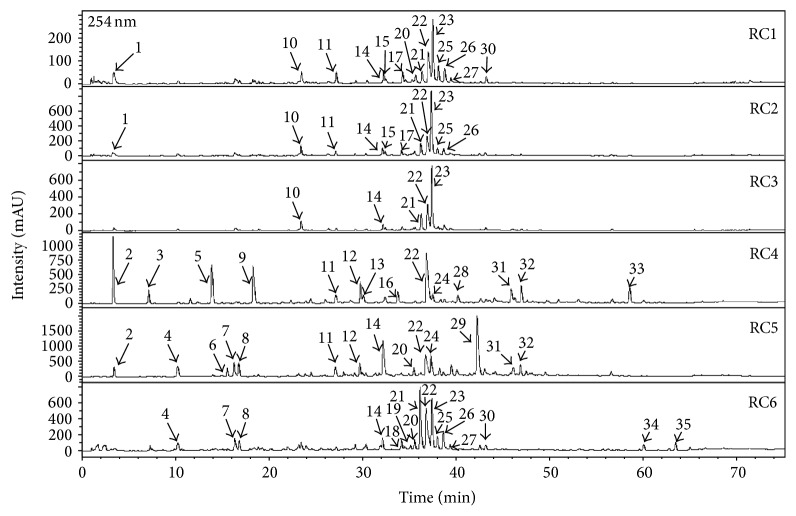
UHPLC chromatograms of* R. caesius* samples (RC1–RC6) recorded at 254 nm.

For the first time, we initiated a detailed phytochemical analysis of secondary metabolites and confirmed the presence of derivatives of quercetin and kaempferol, as well as ellagitannins [1, 11]. The fingerprints of the analysed* R. caesius* extracts were established using the HPLC-DAD-MS^3^ method. The analysis revealed the presence of thirty-five constituents (Figure 1) comprising ellagitannins and their derivatives, phenolic acids, as well as flavonoids. In the RC1 (water), RC2 (50% methanol), and RC3 (methanol) extracts ellagic acid [**22**] and sanguiin H-6 [**23**] were detected as the dominating constituents. The subfractions RC4 (diethyl ether), RC5 (ethyl acetate), and RC6 (*n*-butanol) contained a wide variety of phenolic acids [**2**,** 5**,** 9**,** 11**,** 12**,** 16**,** 22**,** 31 **and** 32**], flavonoids, mainly, quercetin [**18**,** 19**,** 26** and** 34**], and kaempferol [**25**,** 27**,** 30**,** 33** and** 35**] derivatives, as well as ellagitannins and related compounds [**4**,** 6**–**8**,** 14** and** 29**].  Table 1 contains detailed UV-Vis and MS data for all the detected compounds together with their preliminary or full identification. These phytoconstituents express reductive activity on free radicals and may limit the appearance of mutations or even participate in DNA repair [24]. There are a few reports concerning the antitumour activity of other* Rubus* leaves extracts, but no data are available supporting the extracts from dewberry leaves [15, 16, 22, 25, 26]. Previous studies on in vitro models suggest that berry from* Rubus* species may influence colorectal cancer cell survival in concert terms proliferation and apoptosis [17]. Komes and coworkers also revealed that infusion from* R. fruticosus* leaves may induce cytotoxic action against human colon cells, depending of time and concentration [26]. On the other hand, cancer development is closely associated with inflammation and mutatory microenvironments containing free radicals. In another study, a triterpenoid-rich fraction from* R. coreanus* has been shown to express strong anti-inflammatory activity towards injured colonic tissue [27].

**Figure 2:**
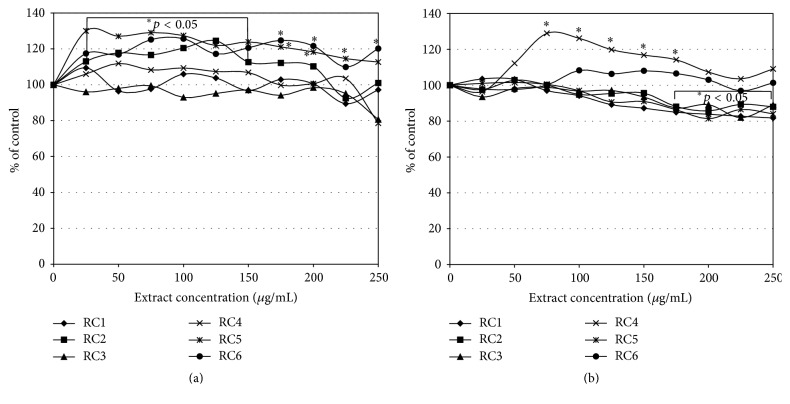
The effect of* Rubus caesius* extracts influence on HT29 (a) and SW948 (b) metabolic activity. The study was conducted for 24 h. The MTT assay. The results are shown as a percentage of the control, arbitrarily set to 100%.

**Figure 3:**
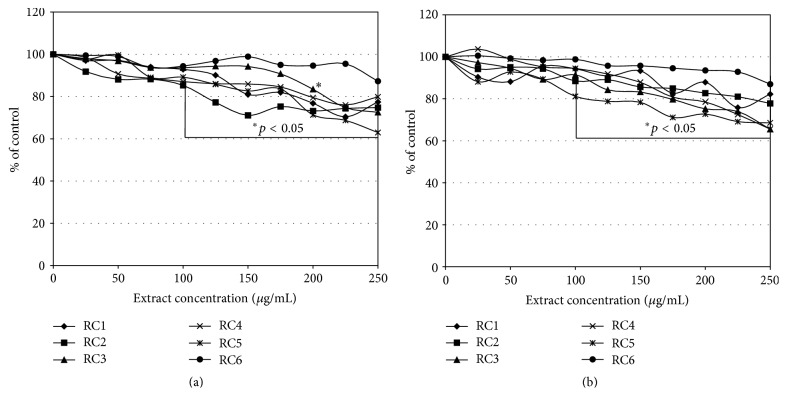
The effect of* Rubus caesius* extracts influence on HT29 (a) and SW948 (b) cellular membranes stability. The study was conducted for 24 h. The NR uptake assay. The results are shown as a percentage of the control, arbitrarily set to 100%.

**Figure 4:**
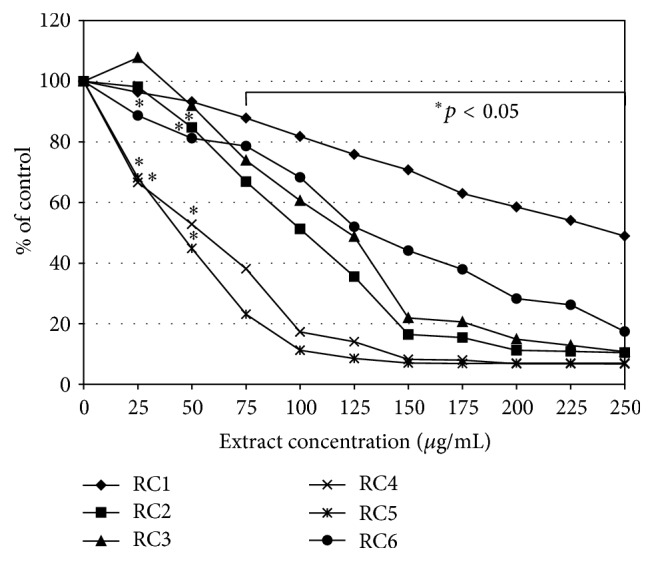
The DPPH free radical scavenging activity. The % of reduced radicals by* Rubus caesius* extracts is compared to the pure methanol activity set as a nonreducing control (0% reduction).

**Figure 5:**
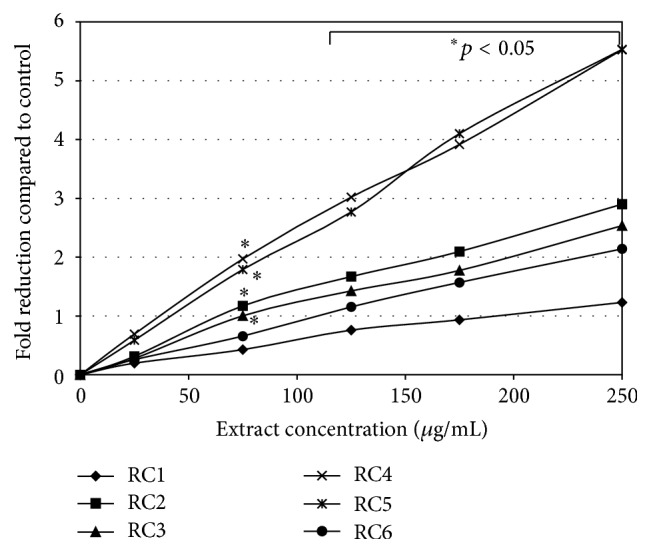
Ferric-reducing activity assay. The % of reduced ferric ions by* Rubus caesius* extracts is compared to the untreated control (0% reduction).

Therefore, we decided to aim our study at the cytotoxic and reduction activity of* R. caesius* leaves in human colon carcinoma cells. Studies on the biological activity of different extracts and subfractions obtained from dewberry were based on two analyses (MTT and NR assays) which were performed on two human colon tumour cell lines HT29 (Duke's A) and SW948 (Duke's C). They were selected to show the reactivity of the early stage of this tumour development. Our study revealed that the tested* R. caesius* extracts expressed no cytotoxic activity. Samples RC2, RC5, and RC6 in a range of concentrations up to 200 *μ*g/mL induced mitochondrial action about 20% above the HT29 cells control (Figure 2). The SW948 cell line was more sensitive to the activity of the tested samples (RC1–3, RC5) than the HT29 line. The inhibitory effect was concentration-dependent. On the other hand, samples RC4 and RC6 induced succinyl dehydrogenase activity. The tested extracts inhibited, in a concentration-dependent manner, the viability of cells of both HT29 and SW948 lines. The most active was RC5, which, applied at the highest concentration (250 *μ*g/mL), decreased the viability of cells (HT29 and SW948) below 66% (Figure 3). The butanolic fraction (RC6), which at the highest concentration did not decrease the viability of cells below 87%, was less active. Our tests revealed that* R. caesius* extracts possess reductive activity. All extracts, with the exception of RC1 at a dose of 250 *μ*g/mL, almost totally reduced DPPH (Figure 4). RC1 at this concentration reduced only half of the radical. Fractions RC4 and RC5 at the lowest concentrations used expressed strong antioxidative activity. IC_50_ values of the tested samples' activity and their comparison to the Trolox action are presented in Table 2. Antioxidant activity of the selected extracts was also determined by the FRAP method, which is based on the analysis of the Fe^3+^ ions reduction ability of the tested compounds. The highest Fe^3+^ ion reduction was shown for the RC4 and RC5 extracts. It was more than 6.5 times higher (at a dose 250 *μ*g/mL) as compared to the control (Figure 5). This result is comparable to 155 *μ*g/mL of ascorbic acid reductive activity. Similarly to DPPH, the lowest reductive action was shown for the RC1 extract. At the highest concentration applied, its activity was only 2.2 times (activity corresponding to 53 *μ*g/mL of ascorbic acid) stronger than the control.

We showed that the tested extracts (RC1–RC3) and subfractions (RC4–RC6) decreased the viability of cells, acting cytotoxically on tumour cells, and simultaneously expressed strong reductive activity. Our results are in agreement with studies by Durgo et al. [25], showing that red raspberry leaf extract expresses cytotoxic and antioxidative effects in the human colon adenocarcinoma (SW480) cell line. This activity was assigned mainly to polyphenolic compounds present in the plant material. Our results also confirmed results of Dai and coauthors, who revealed that the extracts from blackberry significantly limited HT29 human colon tumour cells growth, and the effect was dependent on the concentration applied. This effect was closely connected with the high content of anthocyanins [22]. Moreover, it was shown that acetone extract of* R. fairholmianus* roots influenced human colon tumour cell morphology and reduced their viability via limitation of the intracellular ATP pool and changes in cells' metabolic activity. As a consequence, depleted ATP quantity decreased the tumour cell proliferation rate and stimulated their death, mainly in the apoptotic pathway [28]. Furthermore, extracts from lyophilized fruits of* R. occidentalis* may modulate host immune system processes by impacting on the function and viability of activated human CD4+ and CD8+ T lymphocytes [13]. It may indirectly influence tumour cell development and further metastasis. Analyses in this immunological direction have also been expanded with the use of* R. coreanus* extracts loaded in gelatin nanoparticles. They were used as transport vehicles for the plant extracts and resulted in the significant enhancement of T, B, and NK cells' functionality in all areas of their immune activity [14]. Interesting results using cold water extracts of fresh fruits of* R. caesius* were shown by Turker et al. [29], who found a 100% antitumour efficiency of these extracts on cancer cells. In another study, similar effect was also supported by its antioxidative action with a relatively low IC_50_ value of 5 *μ*g/mL [19]. Similarly, the shoot extracts of* R. idaeus* were found to be a source of sanguiin H-6 and ellagic acid, which exhibit antioxidative as well as cytotoxic activity [18]. Lee and coauthors additionally revealed that sanguiin H-6 induces morphological changes in tumour cells which are similar to apoptotic features. However, this compound does not affect the cancer cell cycle. In general, the molecular pathway of sanguiin H-6 activity is mediated by MAPK p38 and BID cleavage with the participation of caspase-8 [20]. Kim and coworkers have shown that the aqueous extract of the incompletely ripened fruit of* R. coreanum* inhibits cell proliferation and stimulates apoptosis in HT29 cells and that this may be mediated by its ability to activate the caspase-3 pathway [30]. In other study, Bowen-Forbes and coworkers showed that fruit extracts obtained from some* Rubus* species also exhibited great potential to inhibit colon, breast, lung, and gastric cancer cell growth. The authors speculate that the anticancer effect may partially depend on inhibitory action on cyclooxygenase-2 (COX-2) functionality. Moreover, due to high anthocyanin content, it may also strongly influence the oxidative condition in the tumour cell microenvironment [23].

Besides the general activity of* Rubus* extracts on tumour cells, it was shown that the range of such action is based on the horticultural parameters of the plant material. Production factors, both genetic and environmental, determine the usefulness of plants as a material for specific destiny, for example, chemoprevention. Therefore, the degree of inhibition of human colon tumour cell proliferation depends not only on general active phytoconstituents presence, but also on their specific composition which is dependent on cultivar, production site, or stage of maturity [21].

Generally, plant extracts have many biological activities directly aimed at cell morphology and proliferation or indirectly by possessing reductive feature which influence inflammatory state modulating immune system reactivity. In our study* R. caesius* leaf extract revealed tumour cell growth limiting activity, on both the morphology and metabolism levels. Moreover, its antioxidative activity may be connected with colon origin tumour cells growth reduction.

## Figures and Tables

**Table 1:** MS and UV-Vis data of compounds detected in extracts and fractions prepared from leaves of *R. caesius.*

Number	Compound name	Retention time [min]	UV [nm]	[M − H]^−^ *m*/*z*	MS^2^ ions	MS^3^ ions	[M + H]^+^ *m*/*z*	MS^2^ ions	MS^3^ ions	NL detected [amu]
(1)	Gallic acid^s^	3.3	265	169	125b	—	171	**—**	**—**	—
(2)	Unknown phenolic acid	3.4	229, 314	183	139b	—	185	—	—	—
(3)	Unknown compound	7.2	214, 260, 292	363	329, 325, 278, 153b	—	365	333b, 289, 269	—	—
(4)	Unknown ellagitannin	10.2	260	391^a^/783	481, 301b, 377, 311	—	802^c^	785, 767, 483, 465, 303, 277b	—	—
(5)	Methyl gallate^s^	13.9	271	183	125b	—	185	153b	—	—
(6)	Galloyl-HHDP-glucose isomer	15.6	260	633	**481**, 463, 343, 301b, 275	301b, 275	652^c^	482, 465b, 447, 321, 303, 277	—	152
(7)	Unknown ellagitannin	16.3	263	391^a^/783	481, 301b, 377, 311	—	802^c^	785, 767, 483, 465, 303, 277b	—	—
(8)	Galloyl-HHDP-glucose isomer	16.8	261	633	**481**, 463, 343, 301b, 275	301b, 275	652^c^	482, 465b, 447, 321, 303, 277	—	152
(9)	Unknown phenolic acid	18.3	237, 301sh, 321	179	135b	—	181	—	—	—
(10)	Unknown ellagitannin	23.4	260	459	—	—	461	—	—	—
(11)	*p*-Coumaric acid hexoside^t^	27.2	247, 300sh, 325	325	314, 163b, 259	—	355	233, 161b, 135	—	—
(12)	Methyl brevifolincarboxylate^s^	29.9	211, 277, 356	305	273b, 245	—	307	293, 275, 247b, 219	—	—
(13)	Unknown compound	30.2	217, 252, 274, 337	330	327, 287b, 269, 214	—	332	314, 289b, 227	—	—
(14)	Unknown ellagitannin	32.2	261	783^a^/1567	1265, 1103, 933, 633, 301b	—	1591^b^	—	—	—
(15)	Unknown phenolic acid	32.5	218, 310	279	272, 190, 163b	—	281	147b	—	—
(16)	Ellagic acid derivative	33.7	250, 358	331	287b	—	333	315, 289b, 272	—	—
(17)	Unknown ellagitannin	34.1	262	783^a^/1567	1265, 1103, 933, 633, 301b	—	—	—	—	—
(18)	Quercetin rhamnoglucuronide^t^	34.4	255, 263sh, 351	623	321, 301b	—	625	**479b**, 449, 303	303b	146
(19)	Quercetin pentosoglucuronide^t^	35.1	256, 264sh, 351	609	429, 301b, 285	—	611	**479b**, 449, 303	461, 303b	132
(20)	Sanguniin H-2^t^	35.5	263	551^a^/1103	935, 633, 541, 469, 301, 169	—	—	—	—	—
(21)	Ellagic acid pentoside	36.3	251, 354	433	387, **301b**, 161	257b	435	417, 303b, 219	285b	162
(22)	Ellagic acid^s^	37.0	250, 353	301	273, 257b, 245, 187	—	303	295, 285b, 257, 207, 147	—	—
(23)	Sanguniin H-6^t^	37.4	260	934^a^/1870	—	—	—	—	—	—
(24)	Unknown compound	37.5	255, 350	431	269b	—	433	271b, 174	—	—
(25)	Kaempferol rhamnoglucuronide^t^	38.1	265, 347	607	321, 285b, 257	—	609	**463b**, 447, 287, 307, 231	447, 287b	146
(26)	Quercetin 3-*O*-glucuronide^s^	38.8	255, 263sh, 351	477	431, **301b**, 179, 151	273, 257, 179b, 151	479	303b	—	176
(27)	Kaempferol pentosoglucuronide^t^	39.3	265, 343	593	307, 285b	—	595	549, 465b, 353, 287	445, 329, 287b	132
(28)	Unknown compound	40.1	251, 324	309	277, 179, 161b	—	311	—	—	—
(29)	Unknown ellagitannin	42.3	260	558^a^/1117	935, 633b, 483, 459, 301	—				
(30)	Kaempferol 3-*O*-glucuronide^s^	43.1	263, 341	461	415, 285b, 257, 175	267, 257b, 241, 229	463	445, 371, **287b**, 203	258, 241b, 213, 173, 121	176
(31)	Unknown phenolic acid	45.8	230sh, 294sh, 328	359	249, 223, 197, 179, 161b	—	361	163b	—	—
(32)	Unknown phenolic acid	47.0	235, 298sh, 325	503	485, **341b**, 281, 251, 221, 179	323, 281, 271b, 221, 179	504	—	—	162
(33)	Tiliroside^s^	58.5	266, 295sh, 315, 353sh	593	**447**, 307, 285b	327, 285b, 255	595	329, 309b, 287, 235, 217, 165	—	146
(34)	Quercetin derivative	59.9	255, 265sh, 353	533	515, 357, 301b	—	535	303b, 215	—	—
(35)	Kaempferol derivative	63.3	263, 343	663	645, 499, 399, 285b	—	665	**519b**, 379, 287	287b, 159	146

^a^[M − 2H]^2−^,  ^b^[M + Na]^+^, ^c^[M + H2O]^+^, ^t^tentative assignment, ^s^comparisons with chemical standard have been made, b: base peak (the most abundant ion in recorded spectrum), and in bold: ions subjected to MS^3^ fragmentation.

**Table 2:** DPPH radical reduction assay. IC_50_ values of extracts action compared to corresponding activity of synthetic vitamin E derivative (Trolox).

Samples	IC_50_ of extract activity (*μ*g/mL)	Trolox concentration (*μ*g/mL) which corresponds to IC_50_ of extract activity
RC1	240.9	20.5
RC2	101.8	26.8
RC3	123.5	32.7
RC4	54.8	23.0
RC5	44.5	21.3
RC6	131.3	23.0
